# Cyclic Fatigue Life of Two Single File Engine-Driven Systems in Simulated Curved Canals 

**DOI:** 10.22037/iej.v13i1.18037

**Published:** 2018

**Authors:** Mohammad Reza Nabavizadeh, Mahdi Sedigh-Shams, Sara Abdolrasoulnia

**Affiliations:** a *Oral and Dental Disease Research Center, Department of Endodontics, Dental School, Shiraz University of Medical Sciences, Shiraz, Iran;*; b * Department of Endodontics, Dental School, Shiraz University of Medical Sciences, Shiraz, Iran;*; c * Student Research Center Committee, Dental School, Shiraz University of Medical Sciences, Shiraz, Iran*

**Keywords:** Cyclic Fatigue, NeoNiTi, Reciproc, Single-File System

## Abstract

**Introduction:**

This study aimed to evaluate the cyclic fatigue resistance of two single file engine-driven instruments, Reciproc and NeoNiTi, in simulated root canals.

**Methods and Materials:**

Two groups of 15 NiTi endodontic instruments with an identical tip size of 0.25 mm were tested: Reciproc R25 (group A) and NeoNiTi A1 (group B). Cyclic fatigue testing was performed in a stainless steel artificial canal. The simulated canals had a 60^°^ angle and 5-mm radius curvature. The Reciproc instruments were operated using the preset program on torque control electric motor specific for the Reciproc instruments, while the NeoNiTi instruments were operated using the manufacturer recommendation. All instruments were rotated until fracture occurred, and the number of cycles to fracture (NCF) and the length of the fractured tip were recorded and registered. Means and standard deviations of NCF and fragment length were calculated for each system and data were subjected to Student’s *t* test (*P*<0.05).

**Results:**

A statistically significant difference (*P*<0.05) was noted between Reciproc and NeoNiTi instruments. NeoNiTi A1 instruments were associated with a significantly higher mean NCF as compared to Reciproc R25 instruments (833±176 *vs.* 318±87 NCF). There was no significant difference (*P*>0.05) in the mean length of the fractured fragments between the instruments.

**Conclusion:**

NeoNiTi instruments were associated with a significantly higher cyclic fatigue resistance than Reciproc instruments.

## Introduction

Aprerequisite of successful endodontic treatment is maintaining the original shape of the root canal system without procedural accidents [1]. Nickel-titanium (NiTi) instruments have improved the quality of root canal preparation due to their super elasticity, flexibility and cutting efficacy [2]. By using this type of instrumentation, iatrogenic events, such as ledges, zips, perforations and canal transportation, are reduced, and, subsequently, the canal preparation is more satisfactory and predictable [3].

Besides the advantages mentioned above, instrument separation is the main problem during canal preparation with these instruments [1, 4, 5]. They are required to tolerate more movement tension than stainless steel instruments to shape the root canal [6]. Also, NiTi instruments break without previous visible signs of plastic deformation that usually appear before complete instrument separation [7, 8].

Seventy percent of NiTi rotary instrument fractures are due to cyclic (flexural) fatigue [9, 10]. This type of failure happens when the instrument rotates freely in a curved root canal. The part of the instrument shaft that is located on the inside wall of the curvature is compressed and the opposite side is in tension. Numerous cycles of tension and compression are produced at the point of maximum curvature. This leads to metal fatigue and subsequent breaking of the file [11].

**Figure 1:**
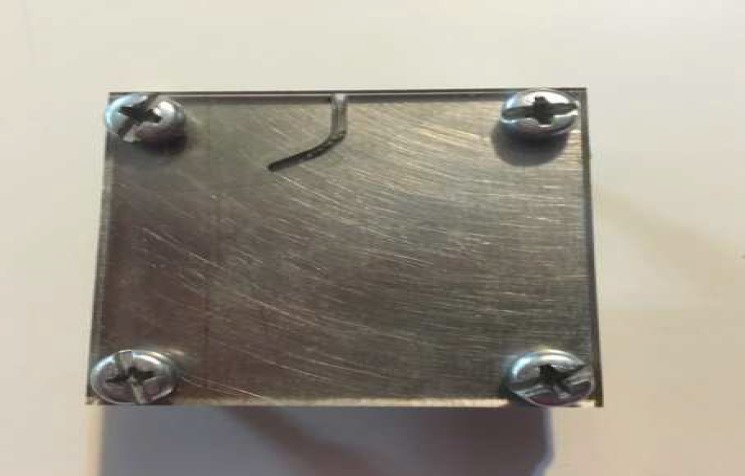
Artificial stainless steel canal

Recently, new instruments with improved NiTi alloys and higher cyclic fatigue resistance (CFR) have become available. Accordingly, introduction of NiTi rotary systems with fewer or even single instrument(s) to the market become possible, and canal preparation becomes faster and easier for practitioners [12]. Single file engine-driven systems are introduced with two types of motion: Complete rotation and reciprocation [13].

Reciproc (VDW, Munich, Germany) is a single file system with an s-shaped cross section that removes root canal dentin by means of reciprocal back (150 degrees counterclockwise) and forward (30 degrees clockwise) motion [14]. The file system is available in three different sizes and tapers: R25 (25/0.08), R40 (40/0.06) and R50 (50/0.05) [15]. 

The files taper discontinuously from the shaft to 3 mm from working length and then taper continuously to the tip. Recommended torque and speed for file movement are 300-500 RPM and 1.5 N.cm [13]. Previous studies have shown acceptable CFR for instruments of this NiTi system [16, 17].

NeoNiTi (Neolix, Charters, la-Foret, France) is another single file system with a non-homogenous rectangular cross section and multiple tapers. The type of motion is a 360-degree rotation [14]. The system consists of an orifice opener, C1 that has a tip diameter of 0.25 mm with a 12% taper at the tip. Three sizes of the A1 file (20#, 25# and 40#) have a taper of 0.08 that prepare the full length of the canal [18].

The instruments are produced by a unique manufacturing process called wire-cut electrical discharge machining (WEDM). In this method, melting, evaporation and ejection of the material are done in a dielectric field. The production of instruments is precise down to microns, clean and oil free, so the stress is limited to the metal surface, and subsequently, the CFR is improved. According to manufacturers, shape memory and elasticity of metal are improved by proper heat treatment, and also, the cutting efficacy is promoted by making the metal surface rough (16); these modifications can also affect CFR. The recommended torque and speed for file movement are 1.5 N.cm and 350 to 550 RPM [18].

In the literature, no comparison between NeoNiTi and Reciproc systems concerning their CFR has been found. The aim of this study was to compare the CFR of these two single file systems.

## Materials and Methods

Using computer-assisted milling, artificial u-shaped cross section grooves were formed in 316L stainless steel blocks to simulate a root canal procedure. The diameter, angle and radius of the curvature of the simulated canal were 1.4 mm, 60 degree and 5 mm. Then the surface was hardened with polished chrome plating. A 4 mm-thick glass was screwed in front of the simulated canal to make the instruments visible in the canal and prevent slippage (Figure 1).

Fifteen of each NeoNiTi A1 (25/0.08) (NEOLIX, Châtres-la-Forêt, France) and Reciproc (25/0.08) (VDW, Munich, Germany) instruments were used in this study.

All instruments were inspected using an optical stereomicroscope under 20× magnification for any signs of visible deformation. All defective instruments were discarded. They were operated by a torque-controlled electric motor (Silver Reciproc, VDW, Munich, Germany) with a room temperature of 24^°^C. NeoNiTi files were operated with a torque of 1.5 N.cm and speed of 350-550 RPM. The Reciproc electric torque control motor was set on “Reciproc All”. The headpiece was fixed above the block to perform the cyclic fatigue test in a reproducible position (Figure 2). Synthetic oil was used to reduce friction between the two metal surfaces of the canal wall and moving instrument. Each instrument was inspected visually, and the time between initiating the test and its fracture was recorded with a 0.01-second precision chronometer.

The number of cycles needed for instrument fracture (NCF) was calculated by multiplying the time recorded in seconds by the number of instrument rotations or cycles per second. Also, the length of the separated instrument was measured to ensure that the instrument was separated at the curvature of the simulated canal.

**Table 1:** Mean (SD) of cyclic fatigue (NCF) (in sec) and fragment length (in mm)

	**Reciproc**	**NeoNiTi**
**NCF**	318 (87^a^)	833 (176^a^176^b^)
**Fragment length**	6.2 (2^A^)	5.9 (1^A^)

Different superscript letters represent statistical significance

The mean length of the fractured segment was also recorded to evaluate if the tested instrument was positioned correctly inside the canal curvature and whether similar stresses were induced. 

Data were subjected to Student’s *t* test with the significance set at the 95% confidence level (*α*=0.05).

## Results

The NeoNiTi group showed greater NCF than Reciproc group (833 and 318, respectively).The difference was significant (*P*<0.001). The NeoNiTi and Reciproc instruments separated at the mean length of 6.2 and 5.9 mm, respectively. There was no significant difference according to fracture length of two groups (*P*=0.4). The mean and standard deviation (SD) for the NCF and fracture length for each instrument are presented in Table 1.

## Discussion

Fracture resistance is important because removing the separated instrument from a canal is difficult, especially when it breaks in the apical third below the canal curvature; if it is not removed, it can have a negative effect on efficacy of cleaning and shaping, as well as the outcome of treatment [12]. This becomes more important when a single file system is used for canal preparation, because the instrument is the first one that introduces in apical third and no cleaning is done before.

Various methods have been used to compare the NiTi rotary files [1, 19, 20]. Some of them, like the present study, determined CFR by operating the instruments in a simulated canal made of a steel block [12, 21]. The main disadvantage of this method is that the file may fit loosely in the canal and more flexibility is allowed for the file at the point of maximum curvature than real canals which results in overestimated data. The advantage of this method is minimizing variables that affect CFR, such as canal diameter and length.

In dynamic models, the axial movement of the handpiece lessens the concentration of strains on one point of the file and improves the CFR [16]. The speed and amplitude of axial motion is standardized in the dynamic model. However, in clinical practice these variables are subjective, and the headpiece movement is controlled manually [21]. In this study, CFR was evaluated during operation of instruments in a static model.

The diameter of the artificial root canal in this study was 1.4 mm; this was to ensure that the instrument had minimal contact with the canal wall as to prevent friction on the lateral aspect of the root canal instrument [22].

The use of standardized artificial canals in cyclic fatigue experiments minimizes the influence of other variables [23]. Similar to other studies, we evaluated CFR of instruments using artificial stainless steel canals with a 60^°^ curvature angle and radius of 5 mm [22, 24-27].

The mean length of the fractured segment was also recorded to ensure the correct positioning of the all tested instruments inside the simulated canal and whether stresses were induced at the curvature of the canal.

Many factors, such as instrument dimensions [12], heat treatment [28], type of metal alloy [29], cross-sectional shape [30], pitch size [31] and helical angle [32] affect the flexibility and CFR of instruments.

We selected Reciproc 25 to compare with NeoNiTi A1, because the sizes of these two instruments are more used for canal preparation than other sizes, especially in small canals. They have the same tip size of 25, also their taper is almost the same (0.08). Also, previous studies showed that Reciproc 25 has acceptable cyclic fatigue compared to other rotary instruments [17, 33, 34].

The speed of instrument movement may also affect the CFR; to eliminate the effect of this parameter, the two instrument groups were operated at the same speed [35].

According to our results, NeoNiTi rotary instruments had 2.6 times more CFR than Reciproc files. Heat treatment adjusts the transition temperature of NiTi alloys, which changes the characteristics of its microstructure and may improve the CFR. Reciproc file system is produced by M-Wire, a kind of heat treatment technology (20). NeoNiTi is manufactured using a kind of heat treatment technique called electrical discharge machining (EDM). All types of conductive materials, including metals and alloys of any hardness, can be manufactured by this technique with high precision [36]. During this procedure, the shape of a work piece is modified by making an electrical potential between the work piece and the tools. The sparks initiated in this process melt and vaporize the material of the work piece in the top layer [37]. One study comparing a NiTi file system benefits from this manufacturing technology showed higher CFR than two instruments manufactured by M-Wire technology [37].

Some studies indicated that reciprocating motion resulted in higher CFRs of endodontic instruments as compared to continuous rotation, regardless of other variables, such as the speed of rotation, the angle or radius of curvature of canals, surface characteristics and geometry of NiTi instruments [16, 22, 38-41]. Reciproc benefits from this type of motion.

Instrumentation geometry (cross-sectional shape, diameters of core, *etc.*) also affects flexibility and CFR. Some studies reported that instruments having the larger cross-sectional area would have more flexural and torsional stiffness [17-19, 42, 43]. This is another reason why the NeoNiTi files with rectangular-like cross sections and more metal mass had better CFR than Reciproc instruments with s-shape geometry.

According to the results of this study, beneficial factors of the NeoNiTi files (processing using EDM technology and geometry of file) improved CFR more than elements that are advantageous for Reciproc instruments (processing using M-Wire technology and type of motion).

More studies are necessary to evaluate cyclic and torsional fatigue of these instruments in different canal lengths and curvatures.

## Conclusion

NeoNiTi® instruments were associated with a significantly higher cyclic fatigue resistance than Reciproc instruments.
